# Cocoa Shell: A By-Product with Great Potential for Wide Application

**DOI:** 10.3390/molecules23061404

**Published:** 2018-06-09

**Authors:** Jelena Panak Balentić, Đurđica Ačkar, Stela Jokić, Antun Jozinović, Jurislav Babić, Borislav Miličević, Drago Šubarić, Nika Pavlović

**Affiliations:** 1 Faculty of Food Technology Osijek, Josip Juraj Strossmayer University of Osijek, Kuhačeva 20, 31000 Osijek, Croatia; jelena.panak@ptfos.hr (J.P.B.); stela.jokic@ptfos.hr (S.J.); antun.jozinovic@ptfos.hr (A.J.); jbabic@ptfos.hr (J.B.); borislav.milicevic@ptfos.hr (B.M.); dsubaric@ptfos.hr (D.Š.); 2 Faculty of Medicine, Josip Juraj Strossmayer University of Osijek, Cara Hadrijana 10E, 31000 Osijek, Croatia; nika.felicita@gmail.com

**Keywords:** cocoa shell, cocoa by-product, bioactive compounds, reuse

## Abstract

Solving the problem of large quantities of organic waste, which represents an enormous ecological and financial burden for all aspects of the process industry, is a necessity. Therefore, there is an emerged need to find specific solutions to utilize raw materials as efficiently as possible in the production process. The cocoa shell is a valuable by-product obtained from the chocolate industry. It is rich in protein, dietary fiber, and ash, as well as in some other valuable bioactive compounds, such as methylxanthines and phenolics. This paper gives an overview of published results related to the cocoa shell, mostly on important bioactive compounds and possible applications of the cocoa shell in different areas. The cocoa shell, due to its nutritional value and high-value bioactive compounds, could become a desirable raw material in a large spectrum of functional, pharmaceutical, or cosmetic products, as well as in the production of energy or biofuels in the near future.

## 1. Introduction

Food industry waste often consists of inedible parts, so-called by-products. Today, there are huge quantities of by-products that are discarded, causing enormous economic problems by polluting the environment. Considering the growing world population and disappearing raw materials, and a real threat of reduced food sources, it is not surprising that awareness about the needs of preservation and re-usage of materials that are treated as a waste is rising [1]. Cocoa shells are just one of the examples of by-products with high-value bioactive components and interesting nutritional value that have been discarded, although they could be re-used in many ways. 

**Figure 1:**
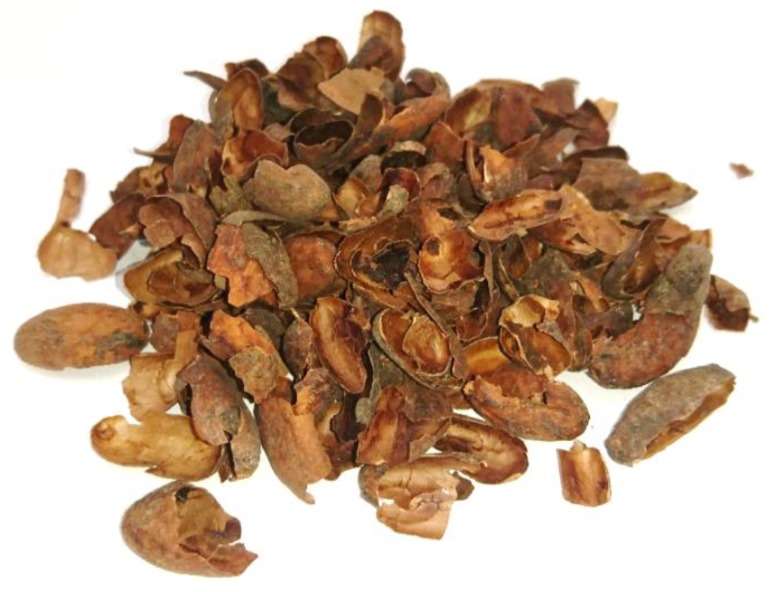
Cocoa shells obtained after roasting beans.

The main raw material for the production of all kinds of cocoa products is dried and fermented cocoa beans, and cocoa shells are one of the by-products of cocoa beans obtained in the chocolate industry. Approximately twenty types of cocoa (*Theobroma cacao*) are known, and the three most popular types (*Criollo*, *Forastero*, and *Trinitario*) make up 95% of the world’s total cocoa production. World production of cocoa beans reached almost 3.7 million tons in 2007/2008 [2]. When cocoa is processed, there are three types of co-products: cocoa pod husk, cocoa bean shells (Figure 1), and cocoa mucilage. 

These by-products are usually considered as “waste” and left to rot on the cocoa plantation, which can cause environmental problems, such as producing foul odors or propagate diseases (e.g., pod rot, because they are not composted) [3,4,5].

**Figure 2:**
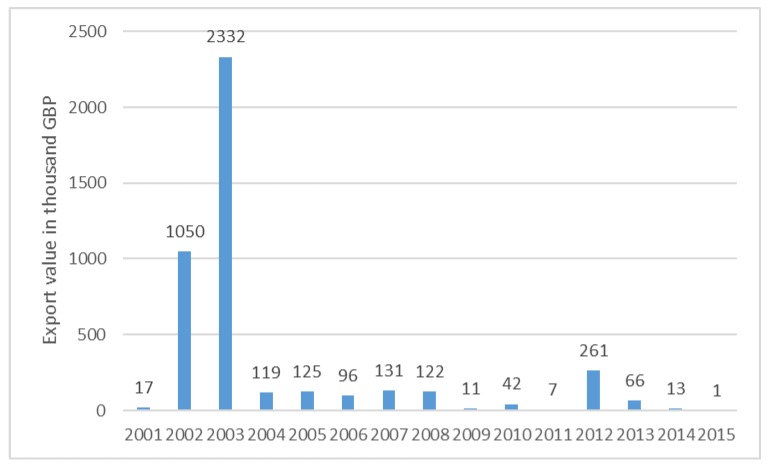
Value of cocoa shells, pod husks, skins, and other cocoa waste exported from the United Kingdom (U.K.) from 2001 to 2015 (in 1000 GBP) [7].

Figure 2 shows statistical data for the value of cocoa shells, pod husks, skins, and other cocoa waste exported from the United Kingdom (U.K.) annually from 2001 to 2015. In 2014, exports of cocoa shells, pod husks, skins, and other cocoa waste were valued at approximately 13,000 British Pounds [6]. Countries in Central and West Africa account for 71.4% of total production, and generate an estimated 6.7 million metric tons of cocoa pod husk, as well as cocoa bean shells and cocoa bean cakes [7]. 

Cocoa shells are the main by-product of cocoa, separated from the cotyledons during the pre-roasting process or after the roasting process [8].

In the Republic of Croatia, there are several chocolate factories and cocoa shells, as a by-product of these factories, are a readily available raw material. Detailed reviews of cocoa shell composition and properties of the most important cocoa shell compounds (polyphenolics, methylxantines, lipids, and fiber) are given by Okiyama et al. [9], thus this paper will focus on further usage of cocoa shells in different areas.

## 2. Cocoa Shell Application

It is possible to use milled cocoa shells, without any modifications, as well as to alkalize cocoa shells, and then use them as a food additive [10].

Relatively high values of dietary fiber [11], together with phenolic compounds, imply that this by-product is interesting to the food industry (in the manufacturing of confectionery products and bakery products, or in the preparation of low calorie dietetic and fiber-rich products, etc.) [12,13]. However, the most common use is still for feedstuff. 

### 2.1. Use in Feedstuff

A number of studies explored the potential of cocoa shells to replace a part of a usual animal diet and investigated their influence on animals, because it contains theobromine, which may have a negative effect on some species [14,15,16]. Specifically, cocoa beans contain approximately 2–3% of theobromine, which crosses from seed into shell during fermentation [17].

The toxicity of a cocoa shell meal to broilers was examined by Day & Dilworth [18]. They added cocoa shell in amounts of 1, 2, 4, and 6% to the meal and concluded that 4 and 6% had a significant influence on the decrease of body weight of broilers. In a subsequent experiment, they added exactly the same amount of pure theobromine as there was in cocoa shells that were in the previous meals, but the broilers’ weight was drastically decreased. Pure theobromine was more toxic than that furnished by the cocoa shell meal. Emiola, Ojebiyi, & Akande [19] confirmed that increasing the intake of sun-dried cocoa shells from 0 to 30% resulted in decreasing average daily feed intake and egg production, together with decreased weight of spleen, kidney, and ovary in hens fed with a diet containing 25 and 30% cocoa shell, because of increased theobromine intake. Olubamiwa et al. [20], however, claimed that cocoa shells that were boiled for 15 min could be used in laying hen feed up to 20% without an influence on egg production and feed conversion.

Adeloye [21] assessed the ability of the goat to utilize some lignocellulosic materials, composed of *Leucaena* leaf meal, yam peels, and cocoa bean shells, together making up 92% of the total diet. This experimental diet had impressive results on weight gain of the goats; 122 and 139 g/day. The feed conversion was 170 g weight gain per kg of feed intake for the female and intact goats, and 200 g weight gain per kg of feed for the castrates. 

Adebowale & Olubamiwa [22] investigated the influence of cocoa shells on growth of *Clarias gariepinus* Burchell, 1822 juvenile. The results indicated that replacement of up to 20% of maize with cocoa shells can favorably support the growth performance of the above-mentioned fish. However, the bitter taste of cocoa shells was one of the major factors that limited its utilization. 

There are other published articles about the usage of cocoa shells for fish diets [23,24,25,26]. In total, there are few other conclusions for juvenile Nile tilapia (*Oreochromis niloticus*) fed with cocoa shells. A cost analysis of diets showed a considerable reduction in the production cost of one kilogram of fish, and feed and nutrient retention efficiencies show that cocoa husk appears to be a viable partial dietary protein source [27].

The economics of using cocoa shells as a food supplement for rabbits was examined by Ayinde et al. [28]. The authors concluded that untreated cocoa shells can be used at 100 g/kg inclusion in rabbit feed, while cocoa shells treated hot water can be included up to 200 g/kg in rabbit feed, for optimum growth performance and the highest cost–benefit ratio. 

Recent studies were oriented on growing pigs. Magistrelli et al. [29] have shown that the use of cocoa shells in pig nutrition may have a positive effect on the balance of intestinal microbial ecosystem. Cocoa shell feeding for three weeks increased microbial populations of the *Bacteroides-Prevotella* group and *Faecalibacterium prausnitzii*, which produce short chain fatty acids, in particular butyrate, which positively influences growth and differentiation of enterocytes, and exerts anti-inflammatory effects, thereby reducing the incidence of a wide range of intestinal inflammatory diseases. Despite a reduction of *Lactobacilli,* cocoa shell feeding improved the proportion between the main phyla of the intestinal ecosystem, which may help to reduce the risk of excessive fattening, which is considered to be detrimental to the quality of the end products [29]. 

Ogunsipe et al. [30] also examined the addition of cocoa shells into pigs’ meals and found that 20% was the optimal biological level of cocoa shells as an energy substitute for maize in a pig diet. 

### 2.2. Use in Agriculture

It is possible to use cocoa husk mulch to suppress weed in perennial fruit crops, gardens, urban landscapes, and occasionally in vegetable crops in organic production systems [31,32].

Arentoft et al. [33] examined the difference between cocoa mulch and bark mulch in suppressing weed growth. Cocoa mulch was more effective, because when compared with bark mulch, a thinner layer of cocoa mulch was needed to reduce the percentage of green pixels by 50% or 90% in relation to control plots. 

Agricultural productivity is impaired in many areas of the world as a result of poor natural conditions and loss of added nutrients that are necessary for plant growth. Hale et al. [34] investigated sorption and desorption of phosphate-P, ammonium-N, and nitrate-N in cocoa shell and corn cob biochars. The authors confirmed that biochar can add and slowly release essential nutrients to soil in order to improve agricultural properties, as the real world biochars used here were able to release PO_4_^3-^–P and weakly exchange NH_4_^+^–N. 

### 2.3. Use in Biofuels

Since fossil fuels present a problem for future generations, there is need for some alternative sources of fuel. Ethanol from lignocellulosic biomass, such as agricultural residue, is one of the important alternatives. In their study, Awolu & Oyeyemi [35] examined ethanol production from cocoa shells using acid hydrolysis and *Saccharomyces cerevisiae*. The result showed that pH had the highest effect on the cocoa shell ethanol yield, followed by fermentation time and yeast concentration; that cocoa shells are an excellent source for such production; and that response surface methodology is a promising tool in the optimization of ethanol production. Additionally, cocoa shells showed good potential for biogas production, with cumulative methane yields [36].

Malatak & Bradna [37] investigated the use of waste material mixtures for energy purposes in small combustion devices. They assessed the energy use of solid biofuels (wheat and rape straw) and their blends with suitable additives (cocoa shells, brown coal, and coal sludge). The results of thermal emission measurements show that all samples meet the requirements of Directive No. 13-2006 [38] for carbon monoxide, but the average nitrogen oxides emission concentrations exceed emission limits. This is because of the high temperature in the combustion chamber and the increasing excess air coefficient. 

### 2.4. Use as an Adsorbent

For the process of adsorption, agricultural waste products are used as natural or modified products through the activation process [39]. Fioresi et al. [40] investigated the grafting of aryl diazonium salt on cocoa shells. The authors concluded that modified cocoa shells can be used as a low-cost adsorbent to entrap pollutants such as heavy metal ions, gases, or industrial dye. 

Bernaet & Ruysscher [41] patented the production of cocoa shell powder free from heavy metals. The cocoa shells have good potential for the treatment of agro-industry wastewaters. They are less effective in removing organic pollutants than the polyethylene material used as a bacterial support. The treatment using cocoa shells produced sludge residues (made up of a mixture of cocoa shells and biomass) containing high amounts of nutrients such as nitrogen, and can be potentially re-used in agriculture as a compost [42].

One way to increase the value of cocoa by-products is by converting the waste into activated carbon. It is suggested as an opportunity to replace the commercial coal-based activated carbon, as it is an eco-friendly product [8]. There are a few different studies about this subject published by Ahmad et al. [43,44,45], Kalaivani et al. [46], Ribas et al. [47], Saucier et al. [48], and Plaza-Recobert et al. [49]. 

Ahmad et al. [43,44,45] showed that cocoa shell-based activated carbons have the potential to be used as an adsorbent for 4-nitrophenol and methylene blue (MB) dye in water or wastewater treatments, and that acid treatment at a higher temperature and higher acid concentration resulted in the development of a new structure of cocoa shell-based activated carbon, which, through the elimination of carbonates and formation of amorphous silica, is highly mesoporous. 

Kalaivani et al. [46] reported that activated cocoa shell carbon (TCAC1 and TCAC2) prepared at two different temperatures (30 and 350 °C) show considerable potential for the removal of Ni(II) ion from an aqueous solution. The heat treatment of the adsorbent has resulted in a smaller particle size and larger surface area and, as a result of the increase in adsorption capacity, shows almost 62% higher efficiency in Ni (II) removal, when compared with TCAC1. 

The application of microwave-assisted activated carbon from cocoa shells as an adsorbent for removal of sodium diclofenac and nimesulide from aqueous effluents was investigated by Saucier et al. [48]. It effectively removed approximately 95% of a mixture of different organic compounds in a medium with high salinity and sugar contents. Plaza-Recobert et al. [49] examined preparation of binderless activated carbon monoliths from cocoa bean husk. The results prove that an adequate combination of the macromolecular components of the cocoa bean husk (lignocellulosic molecules, pectin, gums, and fats), together with a laminate macromolecular microstructure, made it more suitable for obtaining binderless carbon monoliths than other lignocellulosic precursors. Activation of these carbon monoliths gives activated carbon, with larger micropore volume and good mechanical performance.

### 2.5. Use as a Dye

In Tran et al. [50], biofilaments based on cocoa shell waste and biodegradable poly (ε-caprolactonethey are necessary) (PCL) have been prepared using a single-screw extruder. Using this simple and solvent-free fabrication technique, uniformly structured cocoa shell waste biofilaments can be produced in a very reproducible manner, and used in 3D printing of diverse objects with potential household and biomedical applications.

There is also a study that shows that cocoa shell pigment has potential applications as natural dye for fabric dyeing and in the production of UV protective cotton fabric [51].

### 2.6. Use in Food Products

**Figure 3:**
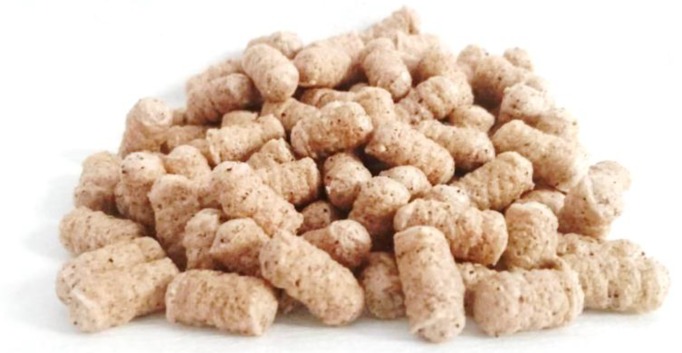
Corn extrudates enriched with cocoa bean shells.

Jozinović et al. [52] produced corn snack products enriched with cocoa shells. They added milled shells to corn grits in 5%, 10%, and 15% d.m. , and extruded in a laboratory single-screw extruder (Figure 3). The authors concluded that it can be successfully employed as nutritional fortification agent. Sanchez Mundo et al. [53,54] used cocoa shell flour for production of muffins and biscuits. 

## 3. Cocoa Shell Extracts

Cocoa shells can also be used as a raw material for the production of extracts rich in fibers, polyphenols, antioxidants, and so on, which can then be used for further applications [55,56,57]. 

The most abundant bioactive compounds in cocoa shell, according to some authors, are shown in Table 1, together with the applied extraction technique.

### 3.1. Polyphenol-Rich Cocoa Shell Extracts

As cocoa shells contain a certain proportion of phenolic components, which are stored in cocoa seed cotyledons, it is believed that they migrate from cotyledon cocoa beans in different chocolate production processes, such as fermentation, roasting, and alkalizing. This reduces the amount of polyphenols in cocoa beans and gives polyphenol-enriched cocoa shells. The most common compounds are flavanols: epicatechin, catechin, and procyanidins. There are a few methods to obtain polyphenol-enriched extracts from cocoa shells. Generally, higher polyphenolic yields are expected from unfermented shells when compared with fermented ones, as well as from roasted shells when compared with unroasted shells.

The majority of published papers are related to classic extraction of cocoa shells using different organic solvents. For example, Hernández-Hernández et al. [58] compared five different extraction methods of cocoa shells, given in Table 1**.** In the first method, cocoa shells were extracted with a methanol/water (80:20) solution at 70 °C for 1 h. The second extraction was made with ethanol/acidified water with HCl at pH 3 (30:70) *v*/*v* by stirring for 2 h at room temperature, followed by filtration and extraction once again, but with a different solution (acetone/water, 70:30, *v*/*v*). The third extraction was done twice with distilled water, by stirring at 70 °C for 1 h. The fourth extraction was done with methanol/acidified water, similar to second extraction method, and the fifth one was done with acidified water extraction, similar to third extraction method. The methanol/acidified water extraction showed the highest results for flavonoid content.

Usually, traditional extraction techniques are very time consuming and require high amounts of solvents and heating, and as a result, there is increasing demand for novel environmentally friendly extraction techniques that give better extract quality and reduce extraction time and solvent consumption. Barbosa-Pererira et al. [59] used pulsed electric field-assisted extraction to obtain extracts with 20% higher recovery of polyphenols (with epicatechin as the main phenolic compound) and methylxanthines than conventional extraction. Mazzutti et al. [61] used integrated green-based processes, combining supercritical CO_2_ and pressurized liquid extraction with ethanol. The extracts with the highest phenolic content were obtained when cocoa shells were previously defatted using supercritical CO_2_ at a pressure of 20 MPa and temperature of 40 °C, and then submitted to pressurized liquid extraction at 10 MPa and 70 °C. Supercritical CO_2_ as a solvent recovers mainly lipids, similar to soxhlet with hexane, and the phenolic content increased in defatted samples. Therefore, the combination of these two extractions, supercritical CO_2_ and pressurized liquid extraction, with ethanol, can be a promising technology to provide two important fractions from cocoa shells, first lipid enriched extracts from SC-CO_2_ and phenolic rich extracts using pressurized liquid extraction.

There are a few studies showing that cocoa shell extracts rich in polyphenols can be used in oral care, because these polyphenols exhibit anti-glucosyltransferase activity. Because of this, the influence of cocoa shells on caries-inducing properties of *mutans streptococci* in vitro, and on experimental dental caries in specific pathogen-free rats infected with *mutans streptococci*, were examined. The results indicate that cocoa shell extract possesses powerful anti-cariogenic potential [62]. Osawa et al. [63] isolated cariostatic substances from the cocoa shell; higher-molecular-weight polyphenolic compounds; and unsaturated free fatty acids, such as oleic and linoleic acids. Higher-molecular-weight polyphenolic compounds showed anti-glucosyltransferase (GTF) activities, and unsaturated free fatty acids showed antibacterial activity against *S. mutans*. 

Kim et al. [64] have patented manufacturing process of glucosyltransferase inhibitors from cocoa shells. The same group of authors [65] found optimal conditions for the recovery of cocoa shells with a high anti-GTF activity, and a high polyphenol content was determined in extracts obtained with 50% aqueous acetone solution at 60 °C for 4 h, followed by fractionation with 50% aqueous ethanol solution using styrene-based resin. 

Matsumoto et al. [66] examined the inhibitory effects of cocoa shell extract on plaque formation in vitro and in vivo. They showed that cocoa shells significantly reduced the adherence of *Streptococcus mutans* to saliva-coated hydroxyapatite, artificial dental plaque formation by *S. mutans*, and the number of *S. mutans* in plaque in vitro. Furthermore, when used as a mouth rinse, the extract significantly inhibited plaque depositions on the tooth surfaces of human subjects. The authors concluded that cocoa shells may be useful for controlling dental plaque formation and subsequent dental caries development in humans. 

The data from Percival et al. [67] show that cocoa polyphenols can inhibit biofilm formation and acid production by *S. mutans*. Venkatesh Babu et al. [68] compared the antimicrobial efficiency of chlorhexidine and cocoa shell extract mouth rinses in children. There was a significant reduction in *Streptococcus mutans* counts in saliva at all follow-up intervals for both mouth rinse groups, but no significant difference in reduction of *Streptococcus mutans* counts in saliva between the chlorhexidine mouth rinse group and cocoa shell extract mouth rinse group. Consequently, it can be concluded that cocoa shell extract mouth rinse can be used in children as an alternative to chlorhexidine mouth rinse, as it has similar antimicrobial properties and evades the side effects of the latter.

Unten et al. [69] investigated whether cocoa shell flavonoids can inhibit cytopathic effects of human immunodeficiency virus (HIV) in cell culture. They have demonstrated that cocoa shell pigment inhibited the replication of HIV in two different assay systems, but the activity of cocoa shell pigment was expressed maximally when cocoa shell pigment was added at the same time as virus adsorption. Cocoa shell pigment is stable against pH and heat, and has no toxicity after oral administration, which are reasons why it has medicinal potential, especially as an antiviral drug [69]. 

### 3.2. Methylxanthine-Rich Cocoa Shell Extracts

During the processing of cocoa beans, in the fermentation stage, methylxanthines migrate from the bean into the shell [17]. Theobromine is the most abundant methylxanthine in cocoa shells, followed by caffeine and teophylline. Theobromine is a white powder; a harsh and odorless component that can be stimulant in moderate amounts, while it may be poisonous in larger amounts. It is characterized by important pharmacological functions, such as anticancer, diuretic, smooth-muscle relaxant, and cardiac stimulants [70]. Theobromine gives bitterness to cocoa and chocolate products [71]. Theobromine also has some antioxidant properties [9,72,73,74]. Arlorio et al. [75] reported 13 g/kg theobromine in dried cocoa shells. According to most studies, teophylline is detected at such low levels that its presence can be ignored. 

Using cocoa shells for animal feed has become questionable because of the high theobromine content [9,71]. This component can have harmful effects on animals if consumed in large quantities, as described earlier in the text. Theobromine can be removed from the cocoa shell by extraction techniques, such as supercritical CO_2_ extraction. It is possible to completely remove theobromine from shells using supercritical CO_2_ and to obtain extracts rich in this component. Hot water treatment has also proved to be capable in reducing the theobromine content [28].

Bradbury & Kopp [57] patented the production of two different extracts from cocoa shells; theobromine fraction and polyphenol enriched fraction. The defatted cocoa shells can be extracted with a solution of acetone/water, after which the acetone needs to be separated, leaving only the aqueous solution. The material is then concentrated, followed by the gel filtration. The theobromine fraction can be rinsed with water, after which a polyphenolic fraction can be rinsed through a column with a low molecular weight solvent.

### 3.3. Fiber-Rich Cocoa Shell Extracts

The proportion of fiber in cocoa shells depends on whether they are roasted or not. It has been reported that, in roasted seeds and shells, formation of Maillard compounds increases the fiber content [76]. The optimal technique for extraction of pectins was given by Mollea et al. [77]. They recommended hot acid extraction in terms of extraction yield, with pH 2.5 and an extraction time of 1 h. Another study shows that the highest yield of pectin (7.62%) was obtained using citric acid at pH 2.5 [1:25 (*w*/*v*)] at 95 °C for 3 h. The highest uronic acid content (65.20%) in the pectin was obtained using water [1:25 (*w*/*v*)] at 95 °C for 3 h [78]. 

Martin-Cabrejas et al. [79] reported a value of 50% for total dietary fiber, while Bonvehi and Beneria [80] determined that total dietary fiber was 57%. Each of these papers reported that the main constituents of insoluble fiber are glucose and uronic acid, with lesser amounts of galactose, arabinose, xylose, and mannose. These sugars indicate that the cell wall polysaccharides in cocoa shell are predominantly cellulose, with lesser amounts of pectin and hemicellulose also present. Redgwell et al. [76] published that total dietary fiber content was approximately 40%, not as high as previous reports. Yeoup Chung et al. [81] found that 100 g d.m. cocoa shell has 26.38 g of lignin, 24.24 g of cellulose, and 8.72 g of hemicellulose.

Although a diet rich in fiber is recommended for preventing and treating constipation, the efficacy of fiber supplements has not been tested sufficiently. A study by Castillejo et al. [82] confirms the beneficial effect of a supplement of cocoa shells that are rich in dietary fiber (39.6 g of total fiber and 13.6 g of ß-fructosans per 100 g of product) on chronic idiopathic constipation in children. 

Cocoa shells have potential health effects on high cholesterol levels as well. This is confirmed by Ramos et al. [83], who reported that the cocoa product obtained after enzymatic treatment of cocoa shells, rich in soluble dietary fiber and with appreciable amounts of antioxidant polyphenols, brought about remarkable hypocholesterolemic and hypotriglyceridemic responses in rats that were fed an atherogenic diet. It also decreased lipid peroxidation, thus diminishing several risk factors for cardiovascular diseases. 

It is also showed that the cocoa shell has nutritional effects, reducing food intake and body weight gain. Another study that shows good potential of soluble dietary fiber is presented by Sanchez et al. [84]. They have been experimenting on rats, where they supplemented their diet with soluble cocoa fiber (SCF) (5%). The results indicate that SCF may modulate parameters that appear altered in the metabolic syndrome, such as body weight, glycemia, insulinemia, lipids, and blood pressure. All of these results show that development of a new source of natural fiber from a waste product from the chocolate industry, such as cocoa shells, could offer a valuable and cheap source of dietary fiber and allow for extensive applications in the food industry. 

## 4. Conclusions

During the processing of raw materials in chocolate industry, a certain amount of by-products is produced, which does not necessarily have to be “waste”, but a by-product or high-value raw material for the development of new products. This review shows that the cocoa shell represents a valuable food industry by-product. Cocoa shells are a rich source of dietary fiber and protein, as well as valuable bioactive compounds (theobromine, caffeine, flavonoids, etc.), and because of their composition, they can be used for further applications as an ingredient in food processing—or in other industries such as pharmaceutical, cosmetic, or agricultural industries—with a constant increase in new applications. In addition, cocoa shell recovery has high economical value, because it is a cheap raw material for the extraction of different components and can be used as biofuel. However, safety of the cocoa shell should be explored more extensively because it is treated with different pesticides, and may contain heavy metals and aflatoxins. 

## Figures and Tables

**Table 1:** The most abundant bioactive components in cocoa shells. n.d.—not determined in the study.

	Hernández-Hernández et al. [58]					Barbosa-Pereira et al. [59]	Arlorio et al. (2001) [60]
Extraction method	Methanol-water extraction	Ethanol-acidified water extraction	Water extraction	Methanol-acidified water extraction	Acidified water extraction	Pulsed Electric Field Extraction	Supercritical CO_2_ extraction
Total phenols (mg/g)	14.64	49.46	5.77	20.39	9.40	24.93 - 32.30	18.2
Theobromine (mg/g)	10.20	11.00	8.47	11.62	6.6	4.64 – 10.92	12.9
Caffeine (mg/g)	n.d.	n.d.	n.d.	n.d.	n.d.	1.59 - 4.21	n.d.
Catechin (mg/g)	1.02	1.97	1.65	4.00	6.16	n.d.	n.d.
Epicatechin (mg/g)	15.84	9.00	6.93	17.70	7.04	0.21 - 2.12	n.d.
